# Characterization of Six-Degree-of-Freedom Sensors for Building Health Monitoring

**DOI:** 10.3390/s21113732

**Published:** 2021-05-27

**Authors:** Louisa Murray-Bergquist, Felix Bernauer, Heiner Igel

**Affiliations:** Department of Earth and Environmental Sciences, Ludwig-Maximilians Universität München, 80539 Munich, Germany; fbernauer@geophysik.uni-muenchen.de (F.B.); igel@geophysik.uni-muenchen.de (H.I.)

**Keywords:** 6DoF, rotation, seismology, SHM, MEMS, FOG, interstory drift

## Abstract

Six-degree-of-freedom (6DoF) sensors measure translation along three axes and rotation around three axes. These collocated measurements make it possible to fully describe building motion without the need for an external reference point. This is an advantage for building health monitoring, which uses interstory drift and building eigenfrequencies to monitor stability. In this paper, IMU50 6DoF sensors are characterized to determine their suitability for building health monitoring. The sensors are calibrated using step table methods and by comparison with earth’s rotation and gravity. These methods are found to be comparable. The sensor’s self-noise is examined through the power spectral density and the Allan deviation of data recorded in a quiet environment. The effect of temperature variation is tested between 14 and 50 ${}^{{}^{\circ}}$C. It appears that the self-noise of the rotation components increases while the self-noise of the acceleration components decreases with temperature. The comparison of the sensor self-noise with ambient building signal and higher amplitude shaking shows that these sensors are in general not sensitive enough for ambient signal building health monitoring in the frequency domain, but could be useful for monitoring interstory drift and building motion during, for example, strong earthquake shaking in buildings similar to those examined here.

## 1. Introduction

Six-degree-of-freedom (6DoF) sensors measure translational motion along three axes and rotational motion around three axes. The collocation of these measurements is an exciting step forward in seismological instrumentation. Translational motion, such as linear displacement, velocity, or acceleration, has long been measured by seismometers, but the addition of rotational measurements, such as rotation angle or rotation rate, bring significant advantages. The collocation of these measurements can provide improved identification of S-waves allowing for decomposition of the seismic wavefield. Furthermore, these sensors can act as a “point seismic array” to determine the speed, direction, and phase of incoming waves [1].

There are currently a variety of approaches to measuring translational and rotational data, each developed for specific applications. In seismology, observatory-based high-sensitivity ring lasers collocated with seismometers have been used to record rotational motion from strong teleseismic earthquakes; these observations improve our understanding of the seismic wavefield [2,3]. In seismic exploration, array techniques have been developed to compute rotational motion [1]. A single 6DoF sensor could provide equivalent rotational information, but there is a trade-off, as the array is also able to provide strain and dilatation measurements between stations [1,4]. Liquid-based rotation sensors are also used; these show potential for measuring strong motions, but they are sensitive to temperature changes [5].

In recent years, the benefits of collocated translational and rotational motion measurements have been noted in structural health monitoring (SHM), an emerging field of engineering that has embraced seismic instrumentation and could further benefit from 6DoF sensors. There are certain requirements that a sensor should meet in order to be useful in SHM. Three requirements are suggested by Bernauer et al. in 2012 for rotation sensors in seismology [5]:

1. Sensors should not be sensitive to linear motion.
2. Sensors should be small and stable with respect to changes in ambient conditions.
3. Sensors should have low enough power consumption to be operable off grid.

A fourth requirement specific to engineering was added by Jaroszewicz et al. in 2016 [6]:

4. The instrument should be able to measure rotational amplitudes up to the order of $\mathrm{rad}\;s^{-1}$ in a frequency range from $10^{-2}$ to 100 Hz.

In this paper, twenty 6DoF IMU50 sensors are examined to determine their applicability to SHM. The sensors use three perpendicular silicon-based capacitive microelectromechanical system (MEMS) accelerometers [7] to measure translational motion, and three Fibre Optic Gyroscopes (FOGs) coiled around the translational axes to measure rotational motion. The FOGs use interferometry to measure rotation rate; interferometry relies on massless photons instead of the motion of a test mass or fluid that would have inertia; thus, the rotational measurements are theoretically independent of translation and should measure pure rotational motion satisfying the first requirement [8,9]. The use of MEMS accelerometers and the tight coiling of the FOGs make these sensors small and compact, satisfying the first part of the second requirement. The stability of these sensors under changing ambient conditions must be tested, but in general both FOGs and MEMS accelerometers have been shown to be stable and robust sensors [5,7,10], so the second requirement is satisfied. These sensors have fairly low power consumption, they were originally designed to be used off-grid for navigation [11], and so the third requirement is met. Finally, the transfer function of a FOG is flat, they generally measure across a wider range of frequencies than that stated in the above requirement, and they have been shown to measure rotational amplitudes on the order of $\mathrm{rad}\;s^{-1}$ [12], so the fourth requirement is also met.

FOGs are a relatively new method of measuring rotation rate, but they have already shown improvement over previous methods of measuring rotational motion. FOGs are already used commercially in navigation [11], and more recently applications for these sensors have expanded into seismology and engineering [4,12]. In marine seismology the collocation of a FOG with an Ocean Bottom Seismometer (OBS) has been shown to increase the signal to noise ratio of the horizontal components of the OBS by removing tilt-contamination [13].

There are various approaches to SHM; in this paper, we will focus on two main methods: monitoring changes in a building’s eigenfrequencies (frequency domain), and monitoring interstory drift (time domain). These methods detect damage such as cracking or weakening that change the stiffness of the structure leading to a shift in the eigenfrequencies [14] and larger amplitude interstory drift [15].

Seismometers and accelerometers have been used to monitor building health for some time [16]. They can monitor the eigenfrequencies of a building and, when multiple sensors are installed, its eigenmodes [17] and interstory drift can be estimated [18]. However, seismometer data, especially in buildings, can be affected by rotations, which could be corrected for with collocated rotation measurements [19]. It has also been shown that rotational mode shapes are more sensitive to damage and provide more information on damage location, which is a major goal in SHM [20,21]. This means that 6DoF sensors could provide a strong advantage in monitoring a buildings eigenfrequencies and eigenmodes, both because the rotation rate information could be integrated and used to correct the accelerometer data for tilt, and because the sensors could simultaneously monitor translational and rotational eigenfrequencies or eigenmodes.

However, to be used for constant monitoring these IMU50 sensors must be sensitive enough to measure ambient building signal, which means the self-noise level of the sensor must be below the amplitude of the ambient building signal. The self-noise of a sensor is the sensor output when there is no input signal. The amplitude of the self-noise can be frequency dependent and relates to the sensitivity of the sensor; signal close to or below the self-noise level can no longer be measured because it is lost in the noise generated by the sensor itself. In this paper, we assess the self-noise levels of 6DoF IMU50 sensors from iXblue and compare the amplitude of the sensor’s self-noise with the amplitude of ambient building noise from two towers: Giotto’s Bell tower in Florence, Italy, and the north tower of the Cologne Cathedral, Germany.

Time domain SHM can be carried out by monitoring interstory drift. Interstory drift can be estimated from seismometer or accelerometer data by integrating to find the difference in displacement between two floors and then using the story height to calculate a tilt angle [18]. Integration of translational data, especially the double integration required for accelerometer data, can be plagued by small offsets that accumulate over the integration [15,22], and as the angle is calculated between sensors the quality of the results is affected by the density of instrumentation [18]. Single integration of the FOG data would provide the same angle directly at the sensor location, removing the need to average over one or more stories, and as the data need only be integrated once this could be a more reliable measurement [12]. Once again, for constant monitoring the self-noise levels of the sensors must be below the ambient building signal levels. However, even low-sensitivity sensors that may not be suitable for constant monitoring could be used to monitor interstory drift during higher amplitude shaking events. To determine the potential of the sensors examined in this paper for this type of monitoring, we will again compare the sensor self-noise levels with ambient building signal levels and with higher amplitude shaking from literature.

These sensors are in principle well suited to SHM. Collocated measurements of translational and rotational motion can provide a full description of building motion without the need for an external point of reference, which can become unreliable, especially during earthquake shaking [5]. The rotation rate data recorded by the FOGs can be integrated to provide the tilt-angle. The tilt-angle provides important information on the building’s interstory drift, and it can be used to correct the MEMS accelerometer data for tilt, which improves the double integration of the data to displacement [19]. Combining these measurements, the sensor orientation and tilt-corrected displacement can be derived; together, they provide a full description of the building’s motion at the sensor location over time, which is a unique advantage of 6DoF sensors. As well, although there are theoretical methods of wave decomposition to estimate the components of rotational and translational motion, there is no reliable way to estimate the complex interaction effects of these motions [23]. 6DoF sensors can directly measure translational and rotational motions, which could be used to model the interaction effects in structures for a more complete description of structural motion. Including 6DoF data, especially rotational motions, in building monitoring unlocks an additional set of observables for SHM. Wavefield gradients such as rotational motions are known to be more sensitive to local structure and heterogeneities [24]. Thus, by including this additional set of observables to SHM (for example, in methods such as coda wave [25] and ambient noise interferometry), we expect improved performance in characterizing material property changes [26,27].

The small size of the sensors makes them easy to install, an advantage in infrastructure monitoring. In addition, both the accelerometers and FOGs are able to measure constant signals, the accelerometers have no AC filter, which makes it possible to use the constant signals of earth’s rotation and gravity as input for calibration, if the position and orientation of the instrument are known. This means that the sensors could potentially be calibrated in situ, which could be used to implement state of health monitoring of the sensors during continuous operation. This further means that the change in gravitational acceleration along the axes of the MEMS accelerometers could be used to calculate the sensor tilt to cross-check the integrated rotation rate data of the FOGs.

The sensors discussed in this paper were provided by the company iXblue to the seismology group at Ludwig-Maximilians University (LMU) Munich to test the potential of such sensors for SHM. The coupling of the more sensitive FOG sensors with relatively cheap MEMS accelerometers is common in navigational instrumentation, but determining whether or not these sensors also fulfill the requirements to be useful in SHM is the focus of this paper. To begin we compare two methods of calibration: the traditional step table method, and calibration using constant earth signals, earth’s rotation rate and gravitational acceleration, as input, Section 3.1. After calibration the basic performance characteristics of the instruments are tested such as sensor self-noise, Section 3.2, and temperature dependency, Section 3.3. The sensor self-noise is examined by computing the Allan deviation and the Power Spectral Density (PSD) of FOG and accelerometer data recorded with no input signal. The sensor self-noise is then compared with ambient building signal, Section 3.4, and examples of larger amplitude signals such as recorded in buildings during earthquake motion and on bridges from traffic noise, Section 3.5. These comparisons allow us to draw conclusions about the potential of these sensors for infrastructure monitoring through eigenfrequencies or interstory drift. This is a major task within the project “Gebäudeschwingungen: kombinierte Zustandsanalyse mit innovativem Sensorkonzept” (GIOTTO), financed by the German Ministry of Education and Science.

## 2. Materials and Methods

The IMU50 sensors examined in this paper have applications in navigation, civil engineering, and seismology [4,11,12]. The use of FOGs gives these sensors an advantage over most other methods of measuring rotation rate as FOGs are theoretically able to measure pure rotation rate. FOGs function by sending light from a beam splitter in two directions around a loop of fibre optic cable coiled around the axis of rotation. The two light beams form a standing interference pattern throughout the cable, when the beams return to the beam splitter they are directed to a photodetector where this pattern is measured [12]. If the coil is rotated, the optical path length of the two light beams changes, changing the interference pattern that arrives at the photodetector [9]. This change in the interference pattern is measured as a phase shift, which can be converted to information on the rotation rate. As the measurements rely on interferometry with mass-less photons instead of the motion of a test mass or fluid with inertia, the rotation rate measurements are independent of translation, and thus should measure pure rotation rate [8,9].

Temperature can change the refractive index of silica, from which fiber optic cables are made [28], and changes in the refractive index of the cable could affect the measured interference pattern. The sensitivity of fibre optic cables to temperature has been exploited to measure temperature in other SHM systems [29]; however, in these sensors the effect could be a disadvantage. In building health monitoring the sensors could see a wide range of temperatures depending on where they are installed in the building and the local weather conditions. Therefore, it is important to test the stability of the sensor measurements with temperature variation. For this purpose, the self-noise of the FOGs and MEMS accelerometers are tested by comparing the PSD of hour-long recordings at temperatures between 14 to 50 ${}^{{}^{\circ}}$C. To assess the stability of the scale or calibration factor of the sensor, one of the FOGs was calibrated with the electric step table over the same temperature range. The MEMS accelerometers require a manual step table for calibration, and therefore could not be calibrated at elevated temperature with the current equipment.

Before the self-noise of these IMU50 sensors can be assessed they must be calibrated. We compare two methods of calibration: the step table method, and calibration using constant earth signals. Absolute calibration is carried out by comparing the sensor output with a known input signal to find the absolute scale factor of the sensor, which is the ratio of the two [30]. Background noise is problematic during calibration as it changes the recorded signal without changing the expected input signal, thereby changing the measured scale factor, and generally increasing the scatter of the scale factors. To mitigate this problem all sensor data was recorded in the relatively seismically quiet environment of a former instrument test platform in the Ludwig-Maximilians University (LMU) Geophysical Observatory in Fürstenfeldbruck.

After calibration the self-noise of the sensors is assessed by examining the Allan deviation and Power Spectral Density (PSD) of recordings of relatively seismically quiet time spans. The self-noise levels of these IMU50 sensors are compared with ambient building signal levels and higher amplitude shaking induced by earthquakes in buildings and traffic on bridges. This comparison is used to assess the potential of these sensors for the different methods of building health monitoring.

### 2.1. Method of Calibration of Rotation Components

The rotation components, measured by FOGs, were calibrated in two ways: using a tilt table and by comparison with earth’s rotation rate. All twenty sensors were calibrated using the tilt table method, the scale factors from this calibration method are shown in Section 3.1.1. For this step a CT-EW1 Step Calibration Table was used [31]. This table is powered by a small motor that performs uniform and stable up–down movements with a micrometer-precision gauge on each side of the table that can be set to zero to measure the relative vertical displacement from the zeroed position. The maximum displacement produced is 1 ± 0.0005 mm as read from these gauges, however, the variation between readings can be up to 0.02 mm. The table comes with a handheld control box so that it can be operated from a meter or two away, which reduces anthropogenic disturbances near the table.

**Figure 1:**
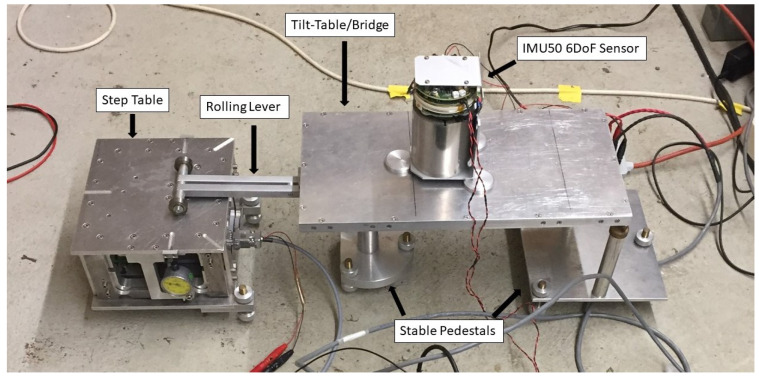
The set up of the sensor on the tilt table for calibration of the FOG components: The step table moves vertically up and down, which tilts the rolling lever, which in turn tilts the bridge or “tilt table” that the sensor sits on. The stationary ends of the rolling lever and tilt table are supported on stable pedestals, all as labelled in the above figure.

To create an input rotation a lever was placed between the step table and a stable pedestal. The lever has wheels on one side allowing it to roll freely on the table as it moves up and down. A secondary stable pedestal was placed nearby with a flat metal bridge spanning the distance between the secondary pedestal and the lever. In this way, the table could move up and down, tipping the lever, which tilted the bridge; this setup is shown in Figure 1.

Steps were made by moving the step table up and down, tilting the sensor by a small angle. Although the electric step table produces uniform steps, the rotation rate is not constant as the table starts and ends in a stationary position. The table does not display the rotation rate or rotational acceleration, but the rotated angle can be calculated from the length of the bridge and the vertical displacement:

(1) $$\theta =\arcsin\frac{ah}{L}$$

where θ is the input angle, *a* is the factor that depends on how far along the rolling lever arm the extension bridge is placed, $\frac{1}{3}$ in this case, *h* is the height the table moves up or down, 1 mm as read from the gauges on the side of the step table, and *L* is the length of the bridge that the sensor is placed on, 405 mm. The resulting rotation angle is $8.23\times 10^{-4}\;\mathrm{rad}$.

**Figure 2:**
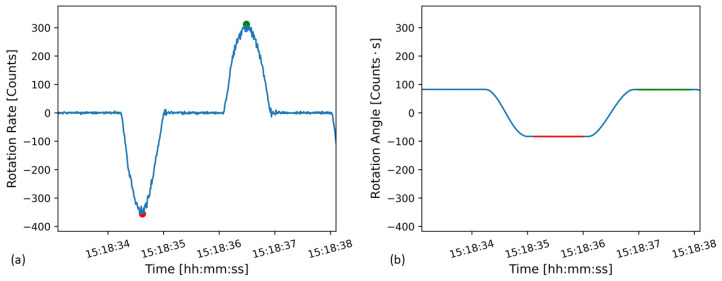
Tilt table output from sensor number 0110 (**a**) direct output showing the sensor being tilted in the negative rotation direction, peak rotation rate in red, and then back, peak rotation rate in green, (**b**) Integrated output showing the rotation angle.

This rotation angle was used to calibrate the FOG sensors. However, as the FOGs measure rotation rate in digital counts, the output must first be integrated once to find the rotation angle with units of count seconds. The direct and integrated sensor output are shown in Figure 2. In Figure 2a the sensor is first rotated in the negative rotation direction, and then rotated in the positive direction back to the starting point. In the integrated data, Figure 2b, this can be seen as a rotation in the negative direction to a constant displacement angle, the red section, and then the rotation angle returns to the initial value, the green section.

The step height recorded by the sensor is then the difference between the red and green flat sections shown in Figure 2b. The locations of the rotation rate peaks, shown in Figure 2a by the red and green dots, were used to help determine the extent of the flat sections in the integrated output. The average of the red flat area was found by taking the average of all points from the index of the peak with the red dot plus 50 to the index of the peak with the green dot minus 50. In this way, the average of the flat areas, which represent the sensor output in between tilt table motions, could be computed. The sensor measured step height is the difference between the integrated data before and after the sensor was tilted, which is the difference between the red and green flat areas in Figure 2b. The buffer of 50 points, corresponding to 0.5 s, around the peak rotation rate is to avoid including sensor output from during the motion when the rotation angle is still changing. This was visually chosen to provide a reasonable balance between excluding points during table motion and including a large number of points for the computed average.

The recorded step height in count seconds is then compared with the input angle in radians to find the scale factor of the sensor in count seconds per radian. As the scale factor is time independent, it can be used to convert the raw rotation rate sensor output from counts to radians per second. To reach a robust estimate of the scale factor 16 steps were recorded in two sensor positions for each rotation axis so that the final scale factor for each axis was computed from 32 steps. The average scale factors and the standard deviations of the scale factors are presented in Section 3.1.1.

The second method of calibration, comparison with the earth’s rotation rate, was tested with one sensor (serial number 0083). Data was recorded in the same basement location of the LMU Observatory from 27 November to 2 December 2019 with a two hour break on 29 November 2019, resulting in two quiet streams of data, hereafter referred to as Stream 1 and Stream 2. This is a low seismic noise environment and so the only input rotation should be earth’s rotation, which is constant, and therefore appears as an offset on the axes. An average scale factor can be found by comparing the offsets of all axes added in quadrature to the total magnitude of earth’s rotation rate. However, the different axes may have unique scale factors, if the exact orientation of the sensor were known this could be calculated separately for each axis using the latitude and orientation. In this case the exact orientation of the sensor was unknown, but the latitude was used to split earth’s rotation rate into vertical and horizontal components and these scale factors were calculated individually.

To find a stable estimate of the scale factor, the stream was split into sections of 6001 points, or 60 s. The scale factor was computed for each section, and then the average scale factor and corresponding standard deviation were computed from all sections. These are compared with the mean scale factor and standard deviation found for the same sensor using the step table method in Section 3.1.1.

### 2.2. Method of Calibration of Acceleration Components

**Figure 3:**
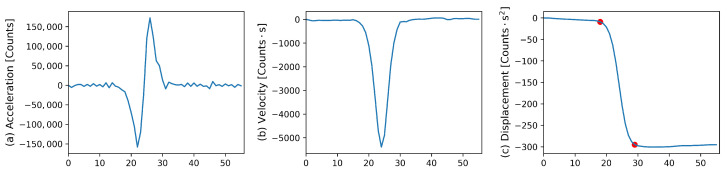
Step table output from one step (step table moving down) with sensor number 0067 (**a**) Direct sensor output (acceleration), (**b**) Integrated sensor output (velocity), (**c**) Twice integrated sensor output (displacement), with manually chosen start and end of step shown by the two red dots.

The MEMS accelerometers were also calibrated in two ways. First all sensors were calibrated using an OSOP brand manual step table. Due to the shape of the sensors, only the HNX and HNZ components could be calibrated. The manual step table was used instead of the electric step table, which was used to calibrate the FOG sensors, as the manual step table can produce higher amplitude accelerations. This is important as the MEMS accelerometer data has higher amplitude noise levels compared to the input signal than the rotation data had, and the accelerometer data must be integrated twice to find the recorded displacement for comparison with the input displacement of 0.662 mm. The sensor recorded step heights were estimated by manually picking the start and end of the step, shown by the red dots in Figure 3c on the twice integrated sensor output. The double integration gives the step height units of counts times seconds squared, this is then compared with the step height in meters leading to a scale factor with units of count seconds squared per meter. The direct sensor output is then corrected with this factor by dividing the output in counts by the scale factor, which results in units of meters per second squared.

The manual steps are less uniform, which, coupled with the required double integration and manual step picking, leads to a higher deviation between steps. The accelerometer scale factors are calculated from the average of 18 steps in two positions aligned with each of the HNX and HNZ axes, so each scale factor is the average of 36 steps. The resulting scale factors and the standard deviations of the scale factors are presented in Section 3.1.2.

As a secondary method, one sensor (serial number 0083) was calibrated using gravity as an input acceleration. The accelerometers can measure constant accelerations, and so earth’s gravity, 9.80782 $m\;s^{-2}$ [32] in Munich, can be seen as a constant offset on the vertical axis of the two quiet streams described in the previous section. As in the calibration of the FOG sensors, the scale factor was calculated for minute long sections by comparing the mean offset to the acceleration due to gravity. The mean scale factor and standard deviation were then calculated from all sections, the resulting scale factor from each stream is compared with the scale factor for the same sensor calculated using the step table method in Section 3.1.2.

The standard deviations given in Section 3.1 represent the over-all uncertainty of the applied calibration procedures. In order to assess the resolution of the sensor measurement we estimate the sensor self-noise that represents the total noise budget of the sensors.

### 2.3. Method of Self-Noise Analysis

The self-noise of these IMU50 sensors was assessed using the two quiet streams of data recorded in the basement test site with sensor number 0083 between 27 November and 2 December 2019. These are the same streams that were used to calibrate the sensor by comparison with earth signals. The former instrument test platform in the LMU observatory is a low seismic noise environment that should have noise levels below the self-noise levels of the sensor. Before analysis, the data were corrected with the scale factors found using the tilt and step table methods. It should also be noted that the sensor data were recorded directly by a laptop, which also provided the timestamps. To avoid data problems due to the low precision of these timestamps, the sampling rate was kept relatively low at 100 Hz. The data were stored as miniseed files and handled using the open-source Python module ObsPy [33].

The type of noise present in the sensors was first assessed in the time domain through Allan deviation, the square root of Allan variance. Allan variance is a way of estimating the frequency stability of a signal in the time domain, and characterizing the noise terms in the signal [34]. The concept was first developed in 1966 by D.W. Allan for characterization of noise in atomic clocks, more recently this method has been adapted for noise characterization of FOGs [35], and has also been applied to MEMS accelerometers [36].

The apparent stability of a signal depends on the time scale examined, a signal may contain high frequency noise but have a constant mean over longer time scales, or a signal may be quite smooth, but with low frequency noise causing long-period oscillations. The type or spectra of noise that dominates the signal depends on the time window examined. Allan variance is a metric for signal stability over different time periods. In this paper we use the “Overlapping Allan Deviation” computed using the Python module Allan Tools [37] by:

(2) $$\sigma_{OADEV}^{2}\left(m\tau_{0}\right)=\frac{1}{2{\left(m\tau_{0}\right)}^{2}\left(N-2m\right)}\sum_{n=1}^{N-2m}{\left(x_{n+2m}-2x_{n+m}+x_{n}\right)}^{2}$$

where $\tau_{0}$ is the interval between measurements, and *m* is an integer such that $\tau =m\tau_{0}$ is the averaging time for which each overlapping Allan variance $\sigma_{OADEV}^{2}$ is computed. The overlapping Allan deviation is then the square root of this, $\sigma_{OADEV}$, *N* is the length of the data series, and *n* is an integer index to sum over the data.

Basically, the Allan variance shows the squared mean difference of the averages of consecutive sections of signal, this makes the Allan variance a function of the averaging time of these sections. Allan deviation is defined as the square root of the Allan variance. A logarithmic plot of Allan deviation as a function of section averaging time τ has linear sections, and the slopes have been shown to be characteristic of different noise spectra [35].

In a similar way the slope of a logarithmic plot of the PSD of a signal indicates the dominant noise type, for example a PSD with a flat mean trend is typical of white noise [35]. The slopes and the corresponding noise types are summarized in Table 1, where α represents the slope of the PSD, and μ the slope of the Allan deviation, both on logarithmic scale plots. If the Allan deviation shows a slope of −1, there is a modified calculation that can be used to distinguish between White angle/velocity noise, and Flicker or Quantization noise, the slopes of the modified Allan deviation plots are listed below under Modified μ [35].

The Allan deviation was plotted as a function of the averaging times τ and linear fitting was used to determine the slope of each linear section. The slopes were then rounded to the nearest half integer to determine the likely noise spectra dominating that time range. The resulting slopes are discussed in Section 3.2.1.

The noise content was also assessed in the frequency domain by computing the PSD of the self-noise data. The PSD was computed using Welch’s method as described in his 1967 paper [39]. PSDs were computed using a segment length of $2^{14}$ points, or 163.83 s, with an overlap of 70%, and Hann windowing. The data was low-pass filtered to 49 Hz, just below the Nyquist frequency at 50 Hz.

The PSD can be used to compare the relative amplitudes of signals in the frequency domain and examine a signal’s frequency content. As discussed above, the slope of a logarithmic plot of a PSD of noise data can be used to characterize the dominant noise type in the frequency domain [38]. The characteristic slopes are shown in Table 1.

### 2.4. Method of Testing Temperature Effects

As previously discussed, changes in temperature could affect the interference pattern measured by the FOG sensors, which could affect the scale factor of the sensor. As the sensors could see a wide range of temperatures depending on the installation location it is important to test the extent of these effects both on the FOG and MEMS sensors.

When testing a seismic sensor for temperature effects it is important to find a seismically quiet way of controlling the temperature. This makes the heating step easier, as light bulbs can be used as a seismically quiet way of increasing the temperature. All temperature tests used the same basic setup. Three light bulbs were placed around the sensor so as to be close to the sensor while touching nothing, especially the wires, which could melt. These light bulbs were controlled via a Conrad temperature control system, which allowed the temperature to be set; this control system included a temperature sensor to inform the system if changes were needed. A brick tower was set close to the step table so that the temperature sensor from the control system and the temperature sensor of a METRAHIT TRMS System Outdoor multimeter could be threaded through holes in the tower to hang close together and near the top of the IMU50 sensor.

A Styrofoam cover was then placed over the setup for insulation. In this way, the temperature could be adjusted to range from the background basement temperature, around 15 ${}^{{}^{\circ}}$C, up to about 50 ${}^{{}^{\circ}}$C.

**Figure 4:**
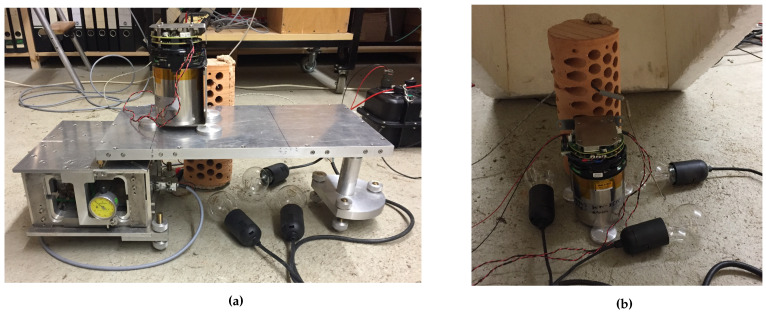
Setup of sensor number 0347 for testing (**a**) the effect of temperature on the scale factor of FOG component HJY and (**b**) the self-noise of the rotation components.

As the FOGs could be calibrated using the electric step table which could be operated with a controller, the effect of temperature on the scale factor of the FOG could be tested with the tilt table. The tilt table setup had to be adjusted slightly to fit under the cover, the bridge was placed directly on the table instead of on the rolling lever arm. The modified setup is shown in Figure 4a. This modification increased the input rotation angle to $2.47\times 10^{-3}\;\mathrm{rad}\pm 3.29\times 10^{-6}\;\mathrm{rad}$.

After the baseline reading, which was generally in the range of 14–15 ${}^{{}^{\circ}}$C, the temperature controller was set and the sensor was allowed to heat for one hour. An hour was seen as adequate based on similar temperature experiments conducted by Bernauer et al. in 2012. In the 2012 experiment the temperature response of other larger FOG sensors was seen to stabilize within an hour, and as the IMU50 sensors explored here are smaller in size, one hour heating time was seen as sufficient [5]. The baseline measurements were always taken with the multimeter before the temperature control system was switched on, then during the heating steps, the temperature of the multimeter and controller were recorded.

To test the effect of temperature on the scale factor of FOG component HJY, sixteen steps were recorded at each temperature step. Unfortunately, the high noise levels of the MEMS accelerometers meant that these required a manual step table for calibration, which could not be operated from under the insulating cover, and so the effect of temperature changes on the scale factor of the MEMS accelerometers could not be tested in this paper. If the sensors are deployed in an area with a wide temperature range this should be tested.

To compare the self-noise of the FOGs and MEMS accelerometers at different temperatures sensor number 0347 was set up as in Figure 4b. Self-noise was recorded at ambient basement temperature and then the controller was set to a temperature and allowed to heat for one hour before recording about an hour of self-noise. For consistency, the recordings were cut to a uniform length of 55 min, and the PSD was computed with a segment length of 40.95 s, and 70% overlap.

### 2.5. Method of Comparison with Building Signal

Data was recorded in Giotto’s Bell Tower, Florence, Italy, between 29 November and 30 November 2016 both on the ground floor and on the top floor at around 82 m height. The towers rotational motion was recorded with a prototype blueSeis-3A rotational sensor from the company iXblue, and translational motion was recorded with a Trillium Compact 120 s seismometer from the company Nanometrics. The Trillium seismometer has a bandwidth of −3 dB points at 120 s and 108 Hz and a dynamic range of 159 dB at 1 Hz [40]. The PSD was computed from hour long sections of recorded data with the instrument response removed. The building signal was recorded with a higher sampling rate of 200 Hz, however for comparison with the IMU50 sensors the PSDs were computed using the same section length and overlap in seconds, that is a segment length of 163.83 s, and an overlap of 70%.

Data was recorded at 100 m height in the north tower of Cologne Cathedral with an updated prototype of the blueSeis-3A sensor to measure rotations, and a Trillium Compact 120 s seismometer to measure translational motion. Data was recorded over several days, but the data used here was taken from 1 November 2017. As the bells toll for about twenty minutes between 8:40 and 9:00 UTC time, twenty minute sections of data were used here to isolate the bell tolling. The same section length and overlap were used to compute the PSDs.

## 3. Results and Discussion

### 3.1. Calibration

It should be noted that the scale factors presented in this section are more accurately named scale divisors: the data is corrected by dividing by the scale factor.

#### 3.1.1. Calibration of Rotation Components

The FOGs were calibrated in two ways; using a CT-EW 01 tilt table from Lennartz Electronics, Germany and by comparison with the earth’s rotation rate. The sensor scale factors and the standard deviations of the scale factors are listed below in Table 2 for each sensor and component calibrated with the tilt table. The scale factors are generally about three orders of magnitude above their corresponding standard deviations, which provide an estimate of the uncertainty of the mean scale factors. The standard deviation of component HJX of sensor number 0025 is an order of magnitude higher than the other standard deviations, this corresponds to the higher noise level seen in this component. The rotation rate around each axis is measured by a separate FOG each with its own connection and wiring, therefore it is possible to have much higher self-noise along one axis than the other two.

There are many sources of error when calculating the scale factor using the tilt table method. There is measurement error, variation in step height, sensor misalignment, and of course any noise including sensor self-noise. As the scale factor is calculated by comparing the integrated data with the angle the sensor was tilted the table’s rotation speed is important. As the table starts and ends in a stationary position the starting and stopping rotational speeds are quite low, and if the rotation speed drops below the sensitivity of the sensor it is possible that the measured step height could consistently underestimate the input angle causing a consistent overestimation of the FOG scale factor. The uniformity of the steps means that this type of error would not affect the standard deviation. However, the sharp peaks of the rotation rate measurements show that the step table accelerates quickly indicating that this is unlikely to be a significant factor, but could be explored further. This error is avoided when calibrating the FOGs by comparison with the Earth’s rotation rate, which is a constant signal that can be directly compared.

The problem of axis misalignment can be seen in the appearance of small steps on axes other than the one being calibrated, although this could also be due to a lack of orthogonality between axes that was not tested. Sensors were aligned on the tilt table manually with lines for guidance, and so the alignment varies slightly each time the sensor is replaced. To mitigate the alignment problem 16 steps from two positions on each axis are used to compute the average scale factor from a total of 32 steps.

The second method of calibration, comparison with earth’s rotation rate, was tested with only one sensor (serial number 0083). The two methods are compared in Table 3. The difference between the two methods is three orders of magnitude smaller than the scale factors, and on the same order of magnitude as the standard deviations, although not always within one standard deviation. These methods are comparable and confirm the correctness of our calibration.

#### 3.1.2. Calibration of Acceleration Components

The MEMS accelerometers were also calibrated in two ways: with a step table and by comparison with gravity. The current shape of the sensors meant that they could not lie on their sides, so only the scale factors of the HNX and HNZ components are shown in Table 4.

The standard deviations are only one order of magnitude smaller than the scale factors. A manual step table was used, see Section 2.2, making the input steps more variable. As well the accelerometer data must be integrated twice to be compared with the input step height, and the sensor recorded step height was manually picked on the twice integrated data, all these factors likely contribute to the higher standard deviations of the scale factors. As discussed in the section above, if the acceleration at some point during the step drops below the sensitivity of the sensor this could lead to some of the displacement of the step not being measured. In the case of the manual step table, the lack of uniform steps means that this may not be a consistent error and so this could add to the larger scatter of the scale factors.

The vertical component of one sensor (serial number 0083) was also calibrated using gravity as an input acceleration. The two methods are compared below in Table 5.

The difference between methods is two orders of magnitude smaller than the scale factors. This is a large difference, but still within the standard deviation of the step table method. The standard deviation of the gravity method of calibration is three orders of magnitude lower than the standard deviation of the scale factor of the step table method, and four orders of magnitude lower than the mean scale factors. The gravity method could have a lower standard deviation because of the stability of the gravity signal, the much larger number of scale factors going into the average, and because the scale factor is calculated by comparing the sensor output directly with the input acceleration instead of having to integrate the output twice, which can accentuate errors. The low deviation of the gravity method scale factors, and that these scale factors are still within the step table standard deviation from the step table value, indicate that this may be a better method of calibration, although it is more time consuming, and in the current sensor orientation only one component could be calibrated at a time. If the sensor could be tilted precisely so that the component of gravitational acceleration along each accelerometer axis were known exactly then all three components could be calibrated simultaneously.

### 3.2. Sensor Self-Noise

#### 3.2.1. Allan Deviation

**Figure 5:**
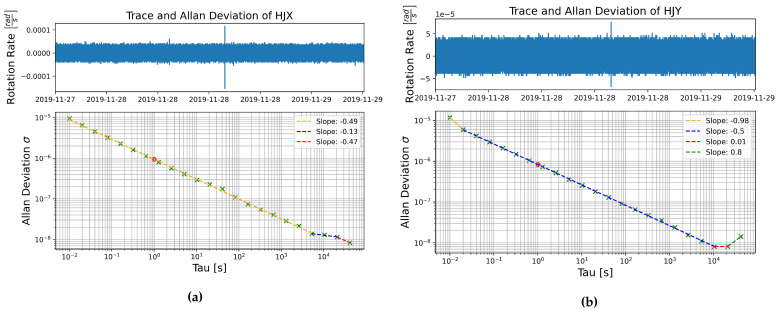
Allan deviation as a function of averaging time τ for self-noise recorded on the FOG rotation components (**a**) HJX and (**b**) HJY, data recorded in the basement test site between 27 November and 28 November 2019.

The Allan deviation was plotted for all components of sensor number 0083 for data Streams 1 and 2. The plots of the FOG components show similar results, the majority of the plot, between averaging times of at least 0.02 s to around $10^{4}$ s, show slopes around −0.5, characteristic of white noise, which, for the FOGs, is caused by angle random walk [35]. Towards longer averaging times the plots plateau to a slope near zero, this is characteristic of pink noise, or bias instability. In the majority of cases, the slope either continues in this plateau or turns upward with a slope of +0.5 or +1, which are attributed to rate random walk and rate ramp, respectively. Rate ramp noise is not really noise as it is a deterministic signal and can be removed with processing. Only in one case, shown in Figure 5a, does the slope return to −0.5 after the plateau.

In two of the components, HJY and HJZ, an initial steeper slope closer to −1 is seen between averaging times of 0.01 and 0.02 s, interestingly this was not seen in either plot of component HJX. An example of this type of plot is shown in Figure 5b. This steeper slope is attributed to noise in the violet spectrum, which can be due to white, flicker, or quantization angle noise. The modified Allan deviation was used to distinguish between these; in this case the modified slope showed that the initial steeper slope of the HJY and HJZ components is likely due to white angle noise.

In general, white noise seems to be the dominant noise source in these FOG sensors. At the shortest of averaging times some violet noise is seen, but only on components HJY and HJZ, and at the longest averaging times bias instability emerges as the dominant noise type.

**Figure 6:**
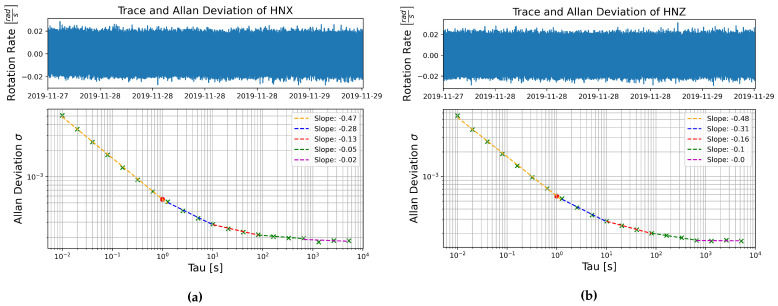
Allan deviation as a function of averaging time τ for self-noise recorded on the MEMS accelerometer components (**a**) HNX and (**b**) HNZ, data recorded in the basement test site between 27 November and 29 November 2019.

The logarithmic plots of Allan deviation as a function of averaging time of the MEMS accelerometer data, Figure 6, are less linear, but a linear slope can be estimated for separate sections. All the MEMS accelerometers showed a linear section with a slope of −0.5, corresponding to white noise, between averaging times of 0.01 s to about 2 s. After 2 s the slope tends to curve as it shallows to a slope of around 0 at averaging times greater than 100 s, this indicates that bias instability becomes dominant at longer averaging times [36].

#### 3.2.2. Power Spectral Density

**Figure 7:**
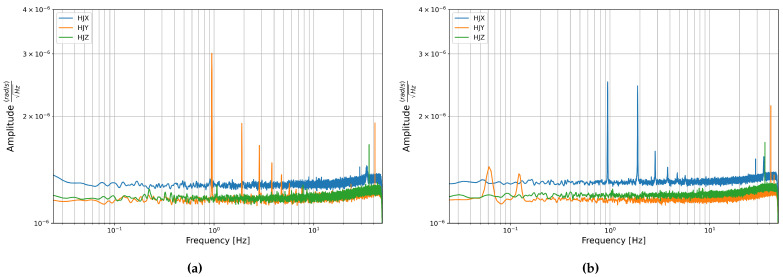
Power Spectral Density of the rotation components from data recorded in the basement test site of the LMU Geophysical Observatory in Fürstenfeldbruck (**a**) Stream 1: from 27 November to 29 November 2019 and (**b**) Stream 2: from 29 November to 2 December 2019.

The PSDs of the rotation components are shown in Figure 7. These are fairly flat across the plotted spectrum with a slight increase in amplitude towards the higher frequencies shortly before the low-pass filter causes a steep decline near 50 Hz. The flat shape of the PSD is characteristic of white noise, which was also the main noise type found in the time domain analysis in Section 3.2.1.

One striking aspect of the PSD of the rotation components is the series of sharp peaks that appear in the HJY component of Stream 1 and the HJX component of Stream 2. In both cases the first peak occurs around 0.95 Hz and the following peaks have an equal spacing of around 0.95 Hz and decrease in amplitude towards the higher frequencies.

This pattern is characteristic of all navigation grade FOGs, and it is caused by “ramp peaks” in the raw data [41]. As the sensors use a closed loop system there is a ramp voltage applied to counter act rotations. This ramp voltage increases and then falls back to zero each time it reaches 2π. The frequency with which 2π is reached depends on the rotation rate the sensor sees. This creates a periodic, but not perfectly sinusoidal signal, which causes the large peak at the main frequency of the signal and harmonics towards the higher frequencies. In navigation these peaks are not a problem as only the integrated signal is used, and the average impact of the ramp signal is mathematically null. However, in seismology the peaks can cause confusion and should be removed. The signal is deterministic and depends on the measured angle of rotation, this information can be output and used to remove the ramp peaks from the raw data, thus removing this peak pattern from the PSDs [41].

Aside from this pattern some shorter but still distinct peaks are visible, these are listed in Table 6. It should be noted that the vertical scale is quite small and so these peaks should not be over-interpreted. Most peaks do not appear in the same place in the two consecutive data streams, and the locations tends to vary between components. Their origin is unknown, but the lack of consistency between components points to random noise rather than some external signal.

**Figure 8:**
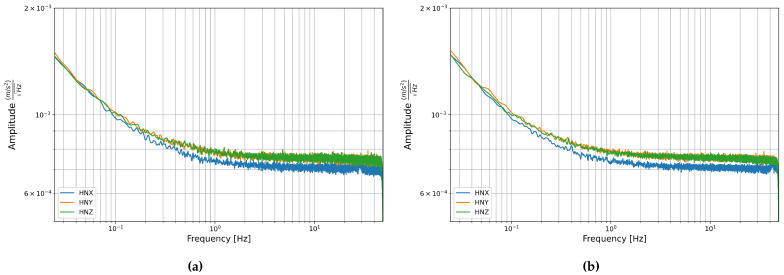
Power Spectral Density of the acceleration components from data recorded in the basement test site of the LMU Geophysical Observatory in Fürstenfeldbruck (**a**) Stream 1: from 27 November to 29 November 2019 and (**b**) Stream 2: from 29 November to 2 December 2019.

The PSDs of the acceleration components are shown in Figure 8. There are no distinct peaks and the main trend is an increase in amplitude towards the lower frequencies and a plateau at frequencies higher than about 1 Hz. It should be noted that the vertical scale is small compared with the amplitude and so this difference in amplitudes between the low and high frequencies is still fairly small.

Linear fitting was carried out on three sections of the PSD: from 0.027 to 0.2 Hz, from 0.2 to 2 Hz, and from 2 to 47.5 Hz. All sections had slopes close to zero, the largest slope appearing in the low frequencies, however, even this only reached about −0.2, which is still in the range of white noise [38]. It appears that similar to the FOGs, white noise is the dominant noise type in the accelerometers. This matches the plots of Allan deviation, which showed a slope of −0.5 indicating white noise at the short periods up to about 1 s, which corresponds to the frequencies above about 1 Hz, which is where we see a plateau in the PSDs. At the lower frequencies, the slope is negative and increases in magnitude towards the low frequencies, which matches the transition to bias instability as the dominant noise type seen in the longer averaging times of the Allan deviation plots.

### 3.3. Temperature Effects

**Figure 9:**
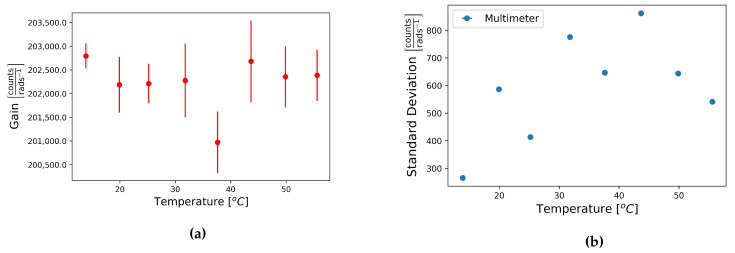
Plots of (**a**) the mean tilt table scale factors and (**b**) the standard deviations of the tilt table scale factors from sixteen steps taken with sensor number 0347 as a function of temperature recorded with the multimeter.

The tilt table scale factor of FOG component HJY of sensor number 0347 was calculated at temperatures between the background test site temperature, around 14 ${}^{{}^{\circ}}$C, and 50 ${}^{{}^{\circ}}$C. These are shown in Table 7, and these results are also plotted as a function of temperature, Figure 9.

No clear trend was visible in the plot of the tilt table scale factor as a function of temperature, however, there appears to be a slight increase in the standard deviation with temperature. To further explore this possible trend the self-noise of each component was recorded at a range of temperatures between 15 and 50 ${}^{{}^{\circ}}$C.

**Figure 10:**
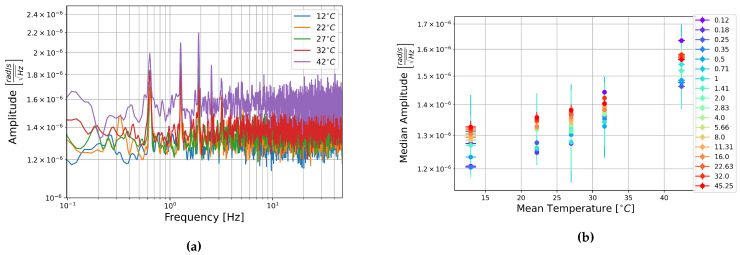
(**a**) Square root of the PSD of component HJX at each temperature step and (**b**) Median amplitude of the PSD per half-octave as a function of temperature. Vertical error bars show STD of amplitudes, and horizontal bars show STD of temperature measurements.

**Figure 11:**
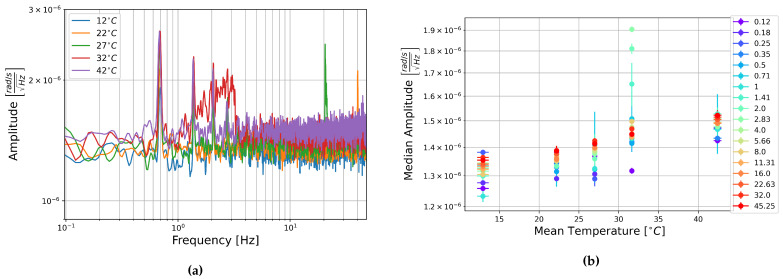
(**a**) Square root of the PSD of component HJY at each temperature step and (**b**) Median amplitude of the PSD per half-octave as a function of temperature. Vertical error bars show STD of amplitudes, and horizontal bars show STD of temperature measurements.

**Figure 12:**
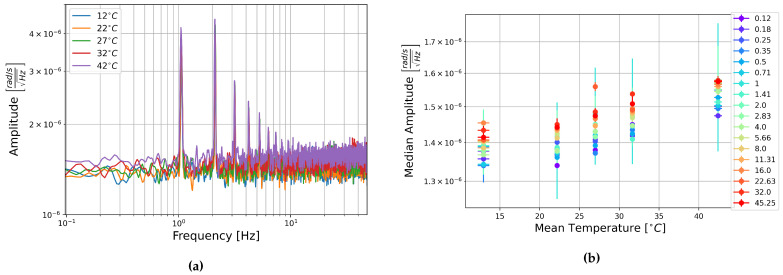
(**a**) Square root of the PSD of component HJZ at each temperature step and (**b**) Median amplitude of the PSD per half-octave as a function of temperature. Vertical error bars show STD of amplitudes, and horizontal bars show STD of temperature measurements.

The square root of the PSDs of the FOG components of these recordings are shown in Figure 10a, Figure 11a, and Figure 12a. These show a general increase in amplitude with temperature across the measured frequency range. All components show the “ramp-peak” pattern discussed in Section 3.2.2. In Figure 11a the PSD of the recording at 32 ${}^{{}^{\circ}}$C makes an extended peak up between about 1 and 4 Hz. It is unclear what causes this higher amplitude section, especially as this is not seen in the other two components.

To represent this another way, the median amplitude of each half-octave of the square root of the PSDs are plotted as a function of temperature. Vertical error bars show one standard deviation of the amplitudes in each half-octave of frequency, and the horizontal error bars show one standard deviation of the temperature as recorded on the multimeter. This is shown for the rotation components in Figure 10b, Figure 11b, Figure 12b. These show an increase in amplitude with temperature, and a general trend towards the higher frequencies having a higher median amplitude. Linear fitting was carried out for the median amplitudes of central frequencies 0.125, 1, 8, and 45.255 Hz. The slopes of these lines of best fit ranged between $4.5\times 10^{-9}$ and $1.5\times 10^{-8}\;\mathrm{rad}\;s^{-1}\;{\mathrm{Hz}}^{-1/2}{\;}^{{}^{\circ}}C^{-1}$. The trend is consistent across all the temperatures measured here, but the low slopes indicate that this is a slight trend. The change in median amplitude per degree Celsius is two to three orders of magnitude lower than the the median amplitudes, indicating that temperature is unlikely to have a strong effect on the data and need not be considered for future analysis of data collected with these sensors.

**Figure 13:**
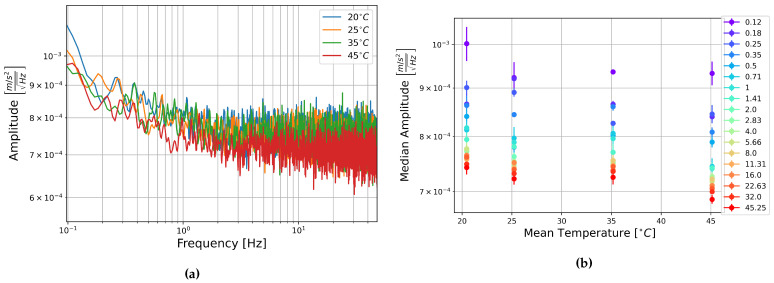
(**a**) Square root of the PSD of component HNX at each temperature step and (**b**) Median amplitude of the PSD per half-octave as a function of temperature. Vertical error bars show standard deviation of amplitudes, and horizontal bars show standard deviation of temperature measurements.

**Figure 14:**
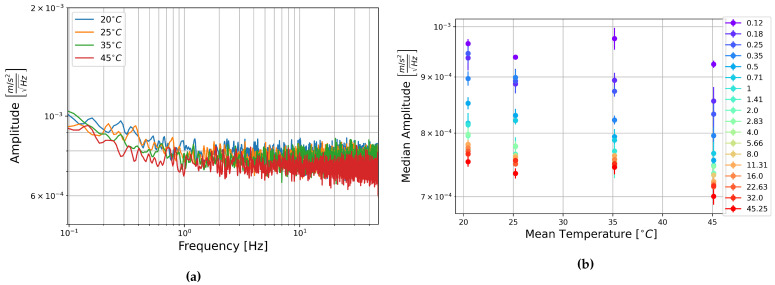
(**a**) Square root of the PSD of component HNY at each temperature step and (**b**) Median amplitude of the PSD per half-octave as a function of temperature. Vertical error bars show standard deviation of amplitudes, and horizontal bars show standard deviation of temperature measurements.

**Figure 15:**
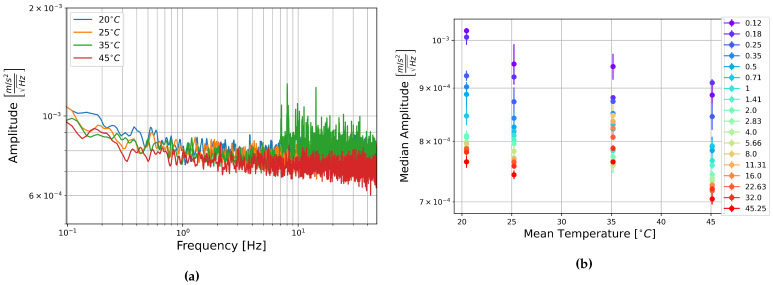
(**a**) Square root of the PSD of component HNZ at each temperature step and (**b**) Median amplitude of the PSD per half-octave as a function of temperature. Vertical error bars show standard deviation of amplitudes, and horizontal bars show standard deviation of temperature measurements.

The square root of the PSD of the self-noise recordings from the MEMS accelerometers at each temperature step are shown next to plots of the median amplitude in each half-octave frequency band as a function of temperature in Figure 13, Figure 14 and Figure 15.

Contrary to the rotation components, it seems that the amplitude per square root Hertz of the self-noise of each component actually decreases slightly as the temperature increases. The plots of the median amplitude as a function of temperature are completely contrary to the trends seen in the rotation components. The median amplitudes are highest in the low frequency bands, decreasing towards the higher frequencies, and the general trend seems to indicate that the median amplitude of self-noise decreases as the temperature increases. One exception can be seen in the plots of component HNZ for the recording at 35 ${}^{{}^{\circ}}$C, which becomes noisy at higher frequencies. It is unclear why this section seems to have such high noise levels, this is not seen in the other two components, and all components show similar amplitudes for the recordings at 25 ${}^{{}^{\circ}}$C and 45 ${}^{{}^{\circ}}$C.

Figure 13, Figure 14 and Figure 15 show that the initial measurement at observatory test site temperature has the highest amplitude, and the measurement at 45 ${}^{{}^{\circ}}$C has the lowest, this appears to be true within error for all components and across the measured frequency range. This result is somewhat surprising as it has been shown that the mechanical transfer function of a capacitive MEMS accelerometer depends on air density, which in turn depends on temperature, and that thermal origin mechanical white noise influences the resolution [42]. It seemed more likely that self-noise levels would increase with temperature, however, details of the construction of these particular capacitive MEMS accelerometers are not known to the authors and there may be other factors that explain this trend.

Once again, linear fitting was carried out for the frequencies 0.125, 1, 8, and 45.255 Hz over the four temperatures tested. The slopes range from $-1.9\times 10^{-7}$ to $-4.65\times 10^{-6}\;m\;s^{-2}\;{\mathrm{Hz}}^{-1/2}{\;}^{{}^{\circ}}C^{-1}$, all but one slope were on the order of $10^{-6}\;m\;s^{-2}$${\mathrm{Hz}}^{-1/2}{\;}^{{}^{\circ}}C^{-1}$. This is about two orders of magnitude below the median amplitudes. It seems that there is a temperature dependence, the self-noise tends to decrease with temperature. However, this is a slight trend, and as in the case of the FOGs, this does not need to be taken into account for further analysis of data from these sensors.

### 3.4. Comparison with Building Signal

To be useful in ambient noise SHM these sensors must have self-noise levels below the ambient building noise. To determine if this is the case the self-noise recorded with one IMU50 6DoF sensor (serial number 0083) is compared with data recorded by a more sensitive FOG rotation sensor and seismometer in two towers: Giotto’s Bell tower in Florence, Italy and the north tower of St. Peter’s Cathedral in Cologne, Germany.

**Figure 16:**
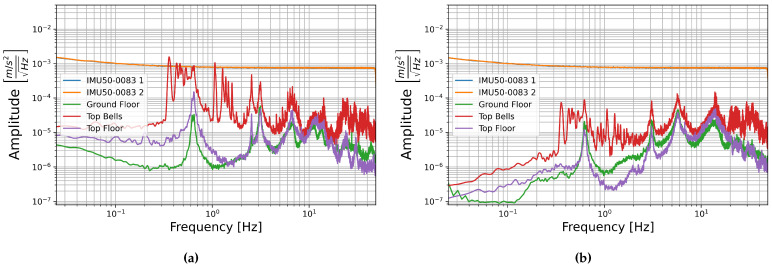
The square root of the PSD of self-noise recordings from sensor number 0083 compared with building noise recorded in Giotto’s Bell Tower on the Ground floor (blue), top floor (orange), and top floor with three bell tolls (green) compared for (**a**) horizontal sensor component HNY with seismometer component HHN and (**b**) vertical sensor component HNZ with seismometer component HHZ.

**Figure 17:**
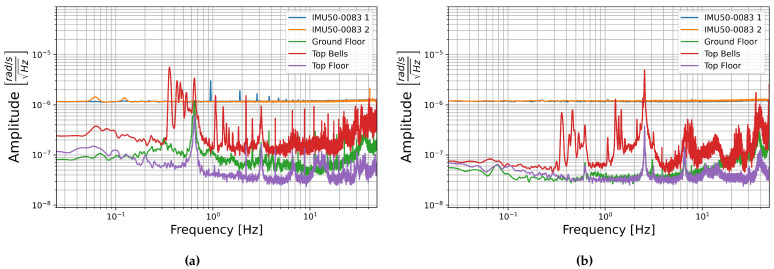
The square root of the PSD of self-noise recordings from sensor number 0083 compared with building noise recorded in Giotto’s Bell Tower on the Ground floor (blue), top floor (orange), and top floor with three bell tolls (green) compared for (**a**) horizontal sensor component HJY with blueSeis-3A component HJ2 and (**b**) vertical sensor component HJZ with blueSeis-3A component HJ3.

Giotto’s Bell Tower is a 84.7 m tall free standing masonry bell tower centrally located in Florence [43]. Three hour long sections were chosen to represent normal building signal levels, one from the bottom floor, one from the top floor, and one from the top floor, which includes three bell tolls; this is likely the high amplitude limit of this buildings signal unless a local earthquake were to occur. The PSDs of these sections are compared with the PSDs of self-noise recorded with sensor number 0083 in Figure 16 and Figure 17.

St. Peter’s Cathedral is centrally located in downtown Cologne, one of the few major cities in Germany with seismic hazard due to its location in the Lower Rhine Embayment [44,45]. Data was collected at 100 m height in the 157.4 m tall north tower, the cathedral’s bells are located at the south tower, and the central train station, which is adjacent to the cathedral, adds to the city noise [45]. Three time spans were chosen for comparison of building signal with the sensors self-noise. The high amplitude limit of building signal is taken to be a twenty minute section from 8:40 to 9:00 UTC time on 1 November 2017 when the Cathedral bells, which are located at the South Tower, ring continuously for All Saint’s Day. The other two sections are limited to twenty minutes to match this record. These are 5:00–5:20, representing low amplitude overnight noise levels, and 15:00–15:20, representing afternoon city noise levels, both are also taken from 1 November 2017 UTC time.

**Figure 18:**
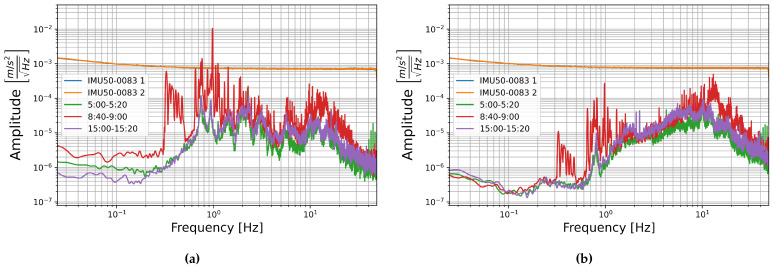
The square root of the PSD of self-noise recordings from sensor number 0083 (blue and orange) compared with building noise recorded in the North Tower of the Cologne Cathedral over three time spans: 5:00–5:20 (green), 8:40–9:00 (red), and 15:00–15:20 (purple) UTC time for (**a**) horizontal sensor component HNX with seismometer component HHE and (**b**) vertical sensor component HNZ with seismometer component HHZ.

**Figure 19:**
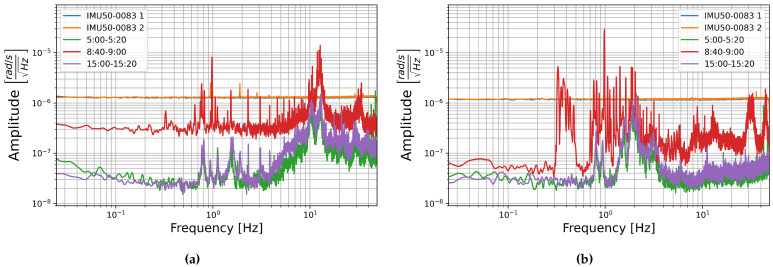
The square root of the PSD of self-noise recordings from sensor number 0083 (blue and orange) compared with building noise recorded in the North Tower of the Cologne Cathedral over three time spans: 5:00–5:20 (green), 8:40–9:00 (red), and 15:00–15:20 (purple) UTC time for (**a**) horizontal sensor component HJX with blueSeis-3A component HJ1 and (**b**) vertical sensor component HJZ with blueSeis-3A component HJ3.

The PSDs of these sections are compared with the self-noise recordings of sensor number 0083 below in Figure 18 and Figure 19. In this case the E-W horizontal components are compared as the bells in Cologne Cathedral swing E-W. As in the case of the building signal recorded in Giotto’s Bell Tower, the amplitude of ambient building signal is generally below the amplitude of both the FOGs and the MEMS accelerometer self-noise amplitudes. Only when the bell ringing induces higher amplitude building shaking does the signal reach into the sensor’s self-noise levels, and then only at certain frequencies.

### 3.5. Comparison with Earthquakes and Active Signals

Higher amplitude shaking can be induced by natural phenomena such as earthquakes, or human induced noise such as traffic or blasting. As has been mentioned in the introduction, seismometers and accelerometers have been used in SHM for some time, and some monitoring systems have been able to record building responses to earthquake shaking. The Van Nuys hotel, located in California, USA, is 20.03 m high and well instrumented with seismometers. Over the course of 24 years at least 11 earthquakes have been recorded with the building instrumentation [16].

**Figure 20:**
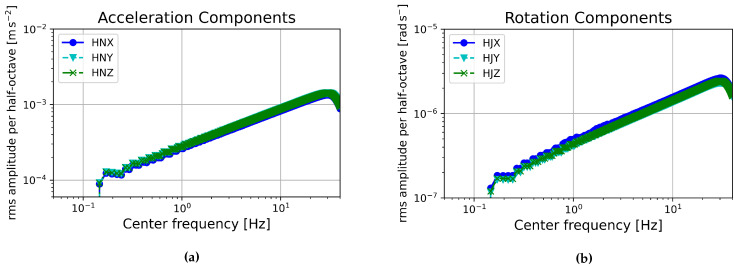
Operating range diagrams showing the root mean squared (rms) power spectral density of sensor self-noise integrated over each half-octave frequency for (**a**) the MEMS accelerometers and (**b**) the FOGs of sensor number 0083 [46].

The recorded earthquakes ranged in local magnitude from 4.1 to 7.5 and in radial distance from 1.5 to 186 km. The resulting peak accelerations ranged from 0.2133 to 4.4222 $m\;s^{-2}$ at ground level and 0.2736 to 5.6320 $m\;s^{-2}$ at the roof [16]. Comparing these accelerations with the Operating Range Diagram (ORD), computed following the description of Evans et al. 2010 [46], of the MEMS accelerometers of sensor number 0083, Figure 20a, the shaking induced by a local medium to large earthquake should be well above the self-noise levels of these sensors. The sensors could therefore be used to monitor building health during earthquake shaking. For this reason, we expect the sensors to be useful in accessing the critical parameter of interstory drift during strong shaking in real time.

FOG sensors are still fairly new and not yet widely used in building monitoring although tests in this area have begun. Shake tables have been used to recreate scaled earthquake sequences to test the suitability of FOGs for building monitoring using scaled building models. One such test of the μFORS-1 FOG sensor predicted peak rotation rates ranging from about $3.5\times 10^{-2}$ to $1.4\times 10^{-1}\;\mathrm{rad}\;s^{-1}$ [47]. Comparing this range with the ORD of these FOG sensors, again computed following the description of Evans et al. 2010 [46] shown in Figure 20b, this should be several orders of magnitude above the self-noise of the sensors and therefore easily measurable. This means the sensors could be used to monitor the rotational eigenfrequencies of the building, and as has been mentioned, the interstory drift of the building could be monitored by integrating the rotation rate data.

Aside from naturally induced high amplitude shaking from earthquakes, traffic is a human source of higher amplitude signal. Although the signal caused by nearby traffic in the buildings looked at was not high enough in amplitude to be used for SHM with these sensors, the traffic signal on a bridge is often of much higher amplitude and could be useful. The amplitude of bridge acceleration depends on various factors such as the span length, material used, and construction style (e.g., suspension, beam type), as well as environmental factors such as wind, and human factors such as traffic conditions [48]. In 1981 a series of tests were carried out on reinforced concrete and steel beam bridges with 1 to 4 spans, and span lengths ranging from 8.2 m to 39.3 m [48]. Accelerometers were used to monitor the crossing of a heavy truck as a test vehicle on these bridges, the mean maximum accelerations from these tests ranged from 0.4064 $m\;s^{-2}$ to 1.8796 $m\;s^{-2}$, with the steel bridges producing amplitudes around twice as high as the reinforced concrete bridges [48]. These are high accelerations, and although these are maximum accelerations, it seems likely that the overall signal would be high enough in amplitude to be recorded by these IMU50 sensors.

The importance of bridge infrastructure has led to SHM programs in some seismically active regions such as the Vancouver area of British Columbia, Canada, a large city with high seismic hazard due to its location in the Cascadia subduction zone [49]. As part of this program seismic sensors were installed on bridges including the Port Mann bridge, a large 2020 m long 65 m wide bridge [50]. This bridge is much longer than those mentioned above and its length and construction material have a strong influence on the magnitude of accelerations it produces. The Port Mann bridge deck is mainly concrete and many of the approach piers use viscous dampers to control bridge motion [50]. It may host larger displacements than shorter bridges, but the size and material likely lower the accelerations. The maximum vertical accelerations measured mid-span in the bridge were plotted over three months and vary between approximately 0.00981 $m\;s^{-2}$ to 0.2943 $m\;s^{-2}$ [50]. The lower range is within the self-noise of the IMU50 sensors, but the upper end of this range should be clearly recorded. The periods with the lowest noise conditions likely occur overnight when both wind and traffic conditions tend to be quieter. The low end of the maximum amplitudes implies that the MEMS accelerometers used in the IMU50 sensors may not be sensitive enough to be used for continuous bridge health monitoring, however, these sensors should be suitable for monitoring bridge structural health during excitation from traffic, strong winds, or seismic events.

## 4. Conclusions

The IMU50 sensors are able to measure constant rotation rates and constant accelerations, this is a unique advantage as the constant signals provided by earth’s rotation and gravity can be used to calibrate the sensors. The calibration scale factors of the FOG sensors were found using the tilt table method and by comparison with earth’s rotation rate. The difference between the FOG scale factors found with the two methods was of the same order of magnitude as the standard deviations and three orders of magnitude smaller than the scale factors themselves, showing that these methods are comparable. The difference between the two methods of calibrating the MEMS accelerometers, the step table method and by comparison with gravity, was only two orders of magnitude smaller than the scale factors, but still within the standard deviation of the step table scale factor. The standard deviations of the MEMS scale factors from comparing the signal with gravity were much smaller, indicating that this could be a better method of calibration, and should be explored further. This method of calibration could also be used to implement state of health monitoring of the sensors to check the scale factors during continuous operation.

The sensors show low sensitivity to temperature variation. The self-noise of the FOGs tends to increase with temperature while the self-noise of the accelerometers tends to decrease with temperature, however, both trends are slight. In the temperature range that these sensors are likely to see for SHM this effect is insignificant, and as the scale factor of the FOG sensors does not appear to be affected by temperature this should not affect the overall performance. The effect of temperature on the scale factor of the accelerometers could not be tested with the available equipment, but this should be tested if the sensors are to be installed outside or in an area where they would be exposed to a wider temperature range.

The comparison of the PSDs of building signal recordings with the self-noise recordings of sensor number 0083 show that ambient building signal generally has lower amplitudes than the sensor’s self-noise. This means that these sensors would not be suitable for ambient noise building health monitoring, however, the sensors could be useful for monitoring building motion during higher amplitude shaking, such as induced by earthquakes. The monitoring of interstory drift by single integration of the FOG data instead of double integration of accelerometer data could increase the quality of interstory drift measurements while requiring fewer sensors making these FOG sensors a powerful new tool in SHM. It has also been shown that traffic can induce high amplitude shaking on some bridges, meaning these sensors could be useful in monitoring the health of certain bridges. These sensors show high potential in the field of infrastructure monitoring, in areas with high signal levels the 6DoF collocated measurements and the ability to theoretically measure pure rotation give these sensors an advantage over previous methods and provide a powerful new tool for public safety.

## Figures and Tables

**Table 1:** Noise types corresponding to the logarithmic slope of the power spectral density, α, and the logarithmic slope of Allan deviation as a function of averaging time, μ [35,36,38].

Noise Type	α	μ	Modified μ
White Angle/Velocity (Violet)	2	−1	−1.5
Flicker Angle/Velocity (Violet)	1	−1	−1
Quantization (Violet)	2	−1	−1
Angle/Velocity Random Walk (White)	0	−$\frac{1}{2}$	—
Bias Instability (Pink)	−1	0	—
Rate/Acceleration Random Walk (Brownian)	−2	$\frac{1}{2}$	—
Rate Ramp	−2	+1	—

**Table 2:** The scale factor and the standard deviation of the scale factor for each FOG. ‘nc’ denotes “not connected” indicating that these components were not operational. All have units of $\left(\mathrm{counts}\;{\mathrm{rad}}^{-1}\;s\right)$.

Sensor Number	HJX		HJY		HJZ	
	Scale Factor	STD	Scale Factor	STD	Scale Factor	STD
0024	200,721	435	200,966	301	200,147	428
0025	200,217	4628	nc	nc	200,848	312
0034	199,494	387	200,442	476	200,171	483
0063	203,097	371	203,509	362	203,308	415
0067	203,236	333	203,915	367	203,835	363
0083	200,689	260	201,082	342	200,499	262
0102	203,186	372	203,291	526	203,281	425
0110	200,747	335	201,010	430	201,373	221
0150	200,972	450	201,112	285	201,004	313
0170	200,221	308	200,544	232	200,961	312
0173	200,653	389	201,282	456	200,948	332
0215	200,968	468	nc	nc	201,126	507
0285	203,263	341	203,265	389	202,118	261
0293	202,397	345	202,092	265	201,595	341
0320	199,464	380	200,215	348	199,812	359
0337	200,083	241	200,136	620	200,408	307
0342	201,549	251	201,954	354	201,915	305
0347	201,673	347	202,045	351	201,436	347
0505	201,542	472	201,663	330	201,618	380
0664	200,885	395	nc	nc	200,982	442

**Table 3:** The mean scale factors of all components combined, and the standard deviations of the scale factors for sensor number 0083 computed from tilt table input and from comparison with the earth’s rotation rate from two quiet streams of data: Stream 1: 27 November to 29 November and Stream 2: 29 November to 2 December, with the difference between these two methods.

Method	Mean Scale Factor $\left(\mathrm{counts}\;{\mathrm{rad}}^{-1}\;s\right)$	Mean STD $\left(\mathrm{counts}\;{\mathrm{rad}}^{-1}\;s\right)$	Difference $\left(\mathrm{counts}\;{\mathrm{rad}}^{-1}\;s\right)$
Tilt Table	200,757	377	—
Earth Rotation: Stream 1	201,460	311	703
Earth Rotation: Stream 2	200,886	315	129

**Table 4:** The scale factors and standard deviations of the scale factors for each accelerometer. ‘nc’ denotes “not connected” indicating that these components were not operational. All have units of $\mathrm{counts}\;s^{2}\;m^{-1}$.

Sensor Number	HNX		HNZ	
	Scale Factor	STD	Scale Factor	STD
0024	410,806	12,414	407,091	14,894
0025	407,031	18,567	406,292	18,360
0034	409,195	26,559	416,221	16,985
0063	nc	nc	411,635	16,505
0067	412,381	16,468	404,720	17,392
0083	411,917	17,381	413,184	18,087
0102	416,146	23,759	412,305	21,807
0110	405,746	22,610	409,390	17,968
0150	415,073	20,759	415,086	15,619
0170	416,082	16,076	413,792	19,552
0173	408,875	19,687	416,936	23,461
0215	nc	nc	409,410	23,759
0285	400,483	22,446	422,534	19,880
0293	423,882	22,223	415,986	15,603
0320	415,662	26,309	414,580	32,066
0337	422,381	23,806	nc	nc
0342	414,306	31,243	406,266	22,794
0347	411,903	17,586	416,931	19,749
0505	407,151	15,565	414,869	17,211
0664	404,975	12,553	407,266	13,235

**Table 5:** The mean scale factors and the standard deviations of the scale factors for sensor number 0083 computed from step table input and from comparison with gravitational acceleration from two quiet streams of data: Stream 1: 27 November to 29 November and Stream 2: 29 November to 2 December, with the difference between these two methods.

Method	Mean Scale Factor $\left(\mathrm{counts}\;{\mathrm{rad}}^{-1}\;s\right)$	Mean STD $\left(\mathrm{counts}\;{\mathrm{rad}}^{-1}\;s\right)$	Difference $\left(\mathrm{counts}\;{\mathrm{rad}}^{-1}\;s\right)$
Step Table	413,184	18,087	—
Earth Gravity: Stream 1	418,708	17	5524
Earth Gravity: Stream 2	418,762	17	5578

**Table 6:** Peak Frequencies (Hz) of the PSDs of streams 1 and 2 for each rotation component.

Component	Peaks in Stream 1 (Hz)		Peaks in Stream 2 (Hz)			
HJX	28.967	34.006	28.955	34.973		
HJZ	1.068	35.938	35.947			
HJY	41.223		0.06104	0.12207	41.223	44.357

**Table 7:** Average temperature measured at each temperature step with the average tilt table scale factors of component HJY and standard deviations of the scale factors.

Date	Multimeter	Scale Factor	STD of Scale Factor
	Temperature (${}^{{}^{\circ}}$C)	$\left(\mathrm{counts}\;{\mathrm{rad}}^{-1}\;s\right)$	$\left(\mathrm{counts}\;{\mathrm{rad}}^{-1}\;s\right)$
21 July 2020	13.9	202,795	265
20 July 2020	15.6	201,767	211
20 July 2020	19.9	202,187	587
21 July 2020	25.2	202,212	413
20 July 2020	31.8	202,275	776
21 July 2020	37.6	200,973	647
20 July 2020	43.7	202,682	862
21 July 2020	49.9	202,356	644
21 July 2020	55.6	202,387	541

## Data Availability

All data and scripts used in this paper are available through LRZ, and can be accessed with this link: https://syncandshare.lrz.de/getlink/fiYW558QkFfoPBMHAyTYZfr1 (accessed on 24 May 2021).

## Abbreviations

The following abbreviations are used in this manuscript:

SHM	Structural Health Monitoring
6DoF	Six Degrees of Freedom
IMU	Inertial Measurement Unit
PSD	Power Spectral Density
ORD	Operating Range Diagram
OBS	Ocean Bottom Seismometer
